# The Mechanisms by Which the Ketone Body D-β-Hydroxybutyrate May Improve the Multiple Cellular Pathologies of Parkinson's Disease

**DOI:** 10.3389/fnut.2019.00063

**Published:** 2019-05-14

**Authors:** Nicholas G. Norwitz, Michele T. Hu, Kieran Clarke

**Affiliations:** ^1^Department of Physiology, Anatomy and Genetics, University of Oxford, Oxford, United Kingdom; ^2^Nuffield Department of Clinical Neurosciences, Oxford University Hospitals NHS Foundation Trust, Oxford, United Kingdom

**Keywords:** apoptosis, dopamine, D-β-hydroxybutyrate, energy metabolism, inflammation, oxidative stress, Parkinson's disease

## Abstract

Parkinson's disease, a progressive neurodegenerative disorder characterized by motor and non-motor symptoms, is strongly associated with the death of dopaminergic neurons in the brain's substantia nigra. Although dopamine replacement therapy temporarily helps patients manage their motor symptoms, this current standard of care fails to address the underlying network of pathologies that contribute to the persistent death of dopaminergic neurons. Thus, new treatment approaches are needed that address the underlying pathologies and, thereby, slow or halt the progression of the actual disease. D-β-hydroxybutyrate – a ketone body produced by the liver to support brain function during periods of starvation – may provide an option. Lifestyle interventions that induce endogenous D-β-hydroxybutyrate production, such as caloric restriction and ketogenic diets, are known to increase healthspan and lifespan in animal models and are used to treat neurological disorders. The efficacy of these ketosis-inducing interventions, along with the recent development of commercially available D-β-hydroxybutyrate-based nutritional supplements, should inspire interest in the possibility that D-β-hydroxybutyrate itself exerts neuroprotective effects. This review provides a molecular model to justify the further exploration of such a possibility. Herein, we explore the cellular mechanisms by which the ketone body, D-β-hydroxybutyrate, acting both as a metabolite and as a signaling molecule, could help to prevent the development, or slow the progression of, Parkinson's disease. Specifically, the metabolism of D-β-hydroxybutyrate may help neurons replenish their depleted ATP stores and protect neurons against oxidative damage. As a G-protein-coupled receptor ligand and histone deacetylase inhibitor, D-β-hydroxybutyrate may further protect neurons against energy deficit and oxidative stress, while also decreasing damaging neuroinflammation and death by apoptosis. Restricted to the available evidence, our model relies largely upon the interpretation of data from the separate literatures on the cellular effects of D-β-hydroxybutyrate and on the pathogenesis of Parkinson's disease. Future studies are needed to reveal whether D-β-hydroxybutyrate actually has the potential to serve as an adjunctive nutritional therapy for Parkinson's disease.

## Introduction

Parkinson's disease (PD) is the world's second most common and fastest growing neurodegenerative disorder. At present, PD affects 2–3% of individuals over the age of 65, a figure that is expected to double by the year 2040 (1, 2). Hence, if it were an infectious disease, PD would quite rightly be called an epidemic. Symptomatically, PD manifests in several classical motor symptoms, including tremors and bradykinesia, as well as in a wide variety of non-motor symptoms, such as disordered sleep and cognitive dysfunction. As there is no cure for PD, symptoms inevitably progress and inflict devastating consequences on individuals and on their families.

In common with Alzheimer and other neurodegenerative diseases, PD is biologically characterized by protein misfolding and the rampant death of neurons. Specifically, PD is characterized by the aggregation of α-synuclein protein and the death of dopaminergic neurons in the midbrain substantia nigra (SN), although PD affects other neurotransmitter systems as well. The pathological mechanisms underlying these biological hallmarks are highly complex and include both metabolic and signaling dysfunctions.

**Figure 1:**
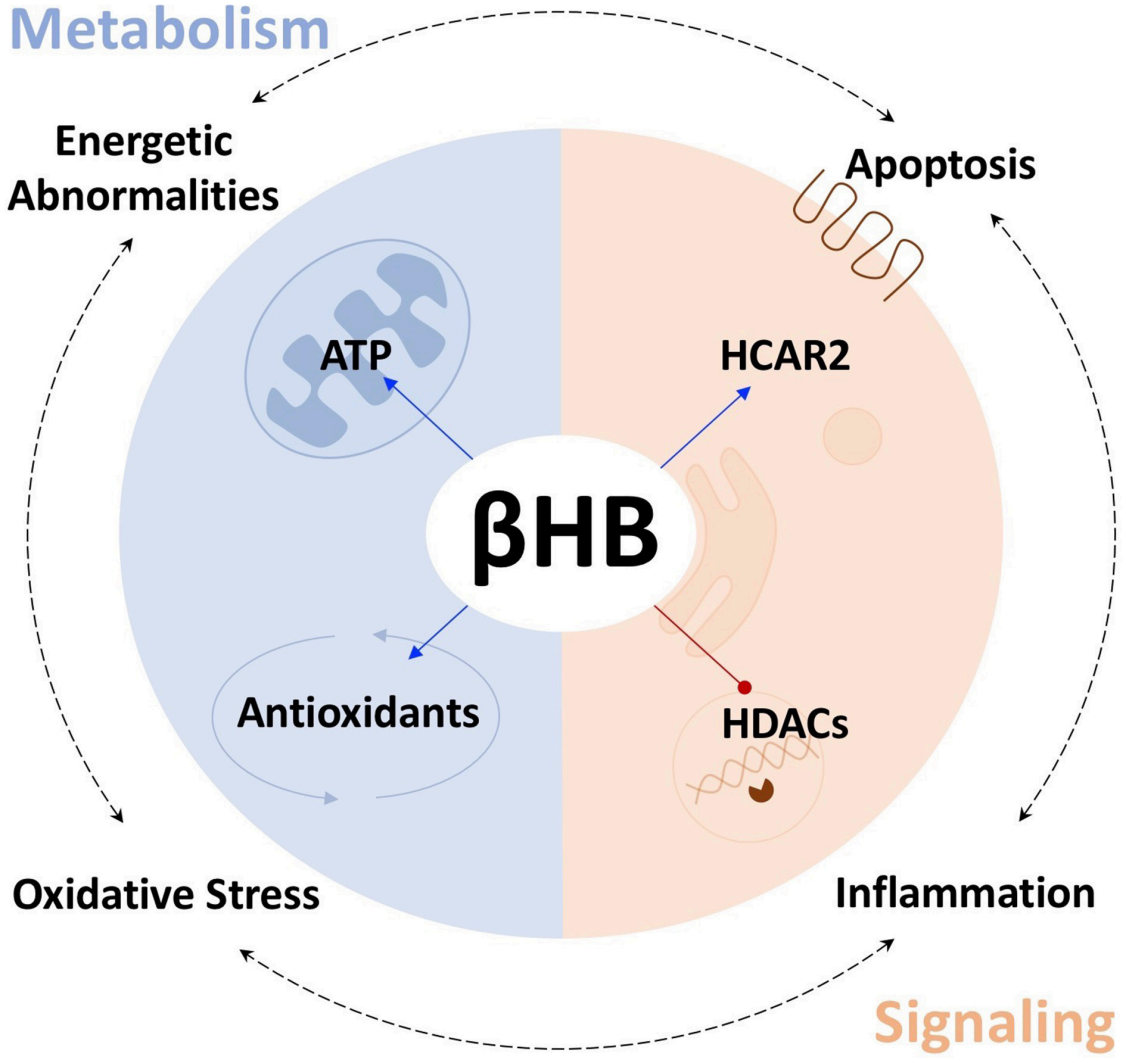
βHB protects against PD pathologies. βHB acts as a metabolite (blue), to increase mitochondrial ATP production and bolster antioxidant defenses, and as a signaling molecule (orange), to activate the G-protein-coupled, hydroxycarboxylic acid receptor 2 (HCAR2), and inhibit class I and IIa histone deacetylases (HDACs), thereby targeting the four fundamental pathologies underlying PD.

As both a fuel substrate and signaling molecule, the ketone body d-β-hydroxybutyrate (βHB) may be well-suited to slow, halt, or even reverse the progression of PD. Indeed, Tieu et al. have shown that that βHB can successfully protect against the death of dopaminergic neurons in the SN and that βHB can alleviate the symptoms of PD in mice (3). At present, many publications are available describing either the ways in which βHB alters metabolism/cell signaling or how βHB affects specific pathological mechanisms that are associated with PD. This review attempts to synthesize these separate bodies of evidence in order to suggest a model for how βHB, acting as both a metabolite and as a signaling molecule, might address many aspects of the pathological network underlying PD; and, therefore, that exogenous βHB supplementation may represent an improvement upon the current standard of care. In this review, we subcategorize βHB's effects on metabolism and cell signaling, respectively, into βHB's effects on (i) ATP production and (ii) antioxidant defenses, and into βHB's actions as (iii) an activator of the G-protein-coupled, hydroxycarboxylic acid receptor 2 (HCAR2) and as (iv) an inhibitor of histone deacetylases (HDACs). Throughout the review we attempt to identify how βHB, acting through these four strategies, can protect against the four most prominent pathological mechanisms in PD, namely abnormal cellular energy metabolism, oxidative stress, inflammation, and apoptosis (Figure 1).

## Abnormal Energy Metabolism

Due to their large size, extensive arborization and calcium-pacemaking activity, SN dopaminergic neurons are particularly metabolically active cells and are, therefore, especially susceptible to energy deprivation (1, 4). Indeed, ^31^P-MRS imaging reveals that ATP levels in the region of the brain that includes the SN are significantly reduced in patients with PD (5). Evidence from mouse models suggests that this relationship is not simply correlative. MPTP, the complex I inhibitor most commonly used to produce animal models of PD, depletes cerebral ATP *in vivo* (6). Furthermore, pharmacologically blocking ATP consumption or increasing ATP production in PD mice is sufficient to prevent α-synuclein aggregation and the death of dopaminergic neurons in the SN, and is sufficient to protect against the motor symptoms of PD (7).

**Figure 2:**
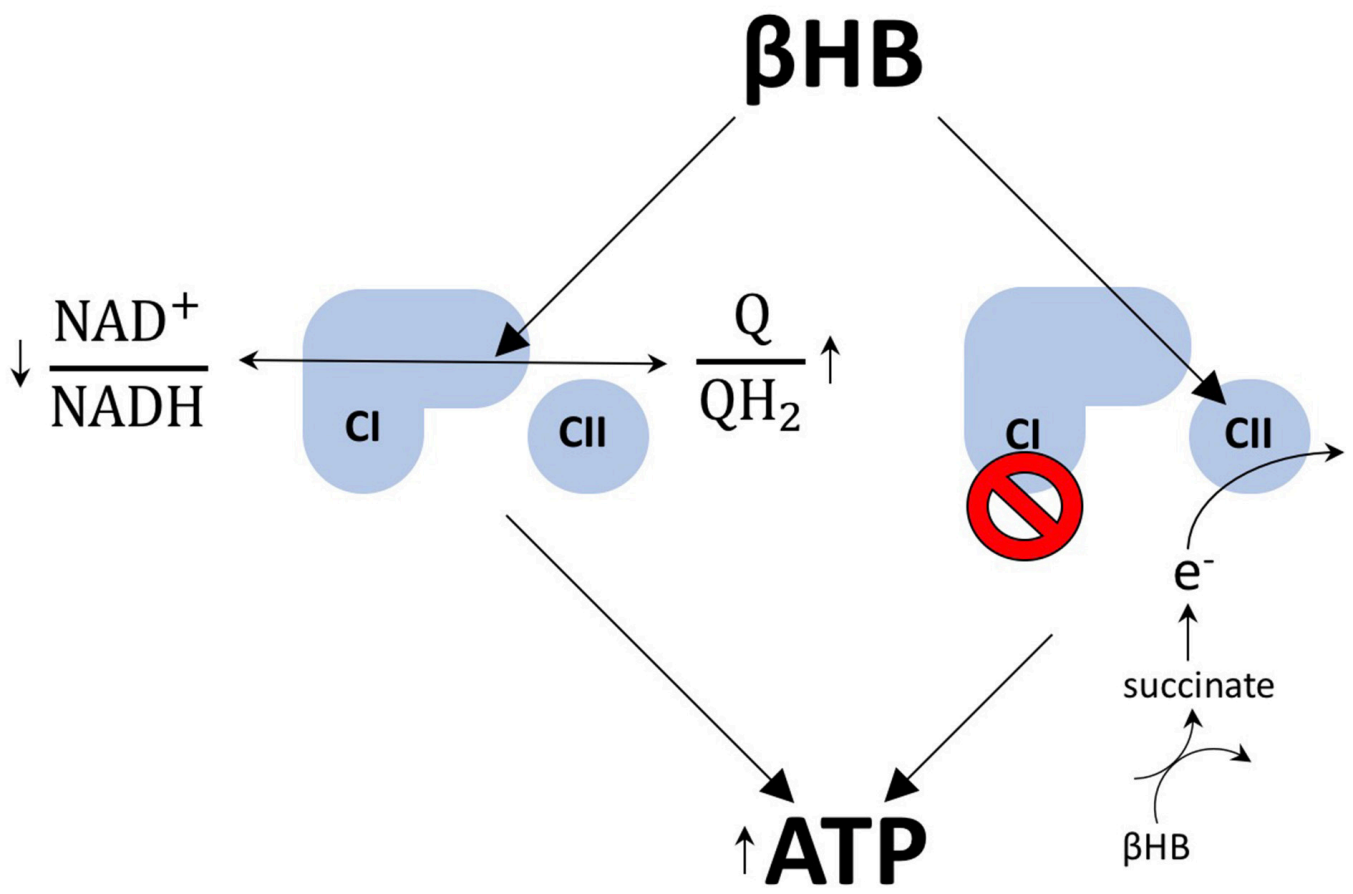
βHB improves energetics. βHB decreases the NAD^+^/NADH ratio and increases the Q/QH_2_ ratio, resulting in an increase in the redox span between the two couples. More energy is liberated by the transfer of electrons from NADH to Q and, thereby, ATP production is increased. βHB also acts to circumvent the pathological blockade of complex I (CI) observed in PD by increasing flux through complex II (CII) via the production of succinate.

There are at least two mechanisms by which βHB may increase ATP levels in dopaminergic neurons. First, the work that launched contemporary scientific interest into the field of exogenous ketones, conducted by Sato et al. on perfused rat hearts, suggests that βHB metabolism increases mitochondrial ATP production by exerting opposite redox effects on the respiratory chain electron carriers, NAD and coenzyme Q (Q). By reducing (decreasing) the NAD^+^/NADH ratio, while simultaneously oxidizing (increasing) the Q/QH_2_ ratio, βHB increases the difference between the redox potentials of these two electron carrier couples (8). This increase in redox span is biochemically analogous to increasing the height span from which a bowling ball is dropped to the ground. In both cases, more energy is available to do work. Therefore, when electrons are passed down from NADH to Q, more protons can be pumped into the intermembrane space to drive the generation of more ATP by chemiosmosis. In this way, βHB metabolism can increase the redox span within the electron transport chain to increase the generation of ATP by oxidative phosphorylation (Figure 2).

Rodent data suggest that βHB metabolism also permits dopaminergic neurons to circumvent the blockade of complex I, a phenomenon that contributes to mitochondrial dysfunction in human PD (9–11), by feeding electrons into the respiratory chain at complex II (Figure 2). This mechanism makes biochemical sense because the rate limiting step of βHB catabolism generates succinate, the oxidative fuel for complex II. Specifically, it has been shown that administration of βHB to MPTP-treated PD mice protects dopaminergic SN neurons from cell death and that this effect is blocked by the specific inhibition of complex II (3). In addition, βHB is able to increase ATP levels in brain mitochondria in the presence of MPTP-mediated complex I inhibition, but not when flux through complex I and complex II are both inhibited (3).

And, although flux through complex II can be linked to a decreased Q/QH_2_ ratio, the redox span and complex II flux models are not contradictory because an increase in flux through a pathway does equate to an increase in the metabolites in that pathway. In fact, βHB only increases succinate levels when flux through complex II is blocked (3), a finding consistent with the notion that βHB can increase Q/QH_2_ turnover without decreasing the ratio itself. Therefore, these two mechanisms, whereby βHB increases ATP production in PD, may be less contradictory than complimentary.

## Oxidative Stress and Antioxidants

**Figure 3:**
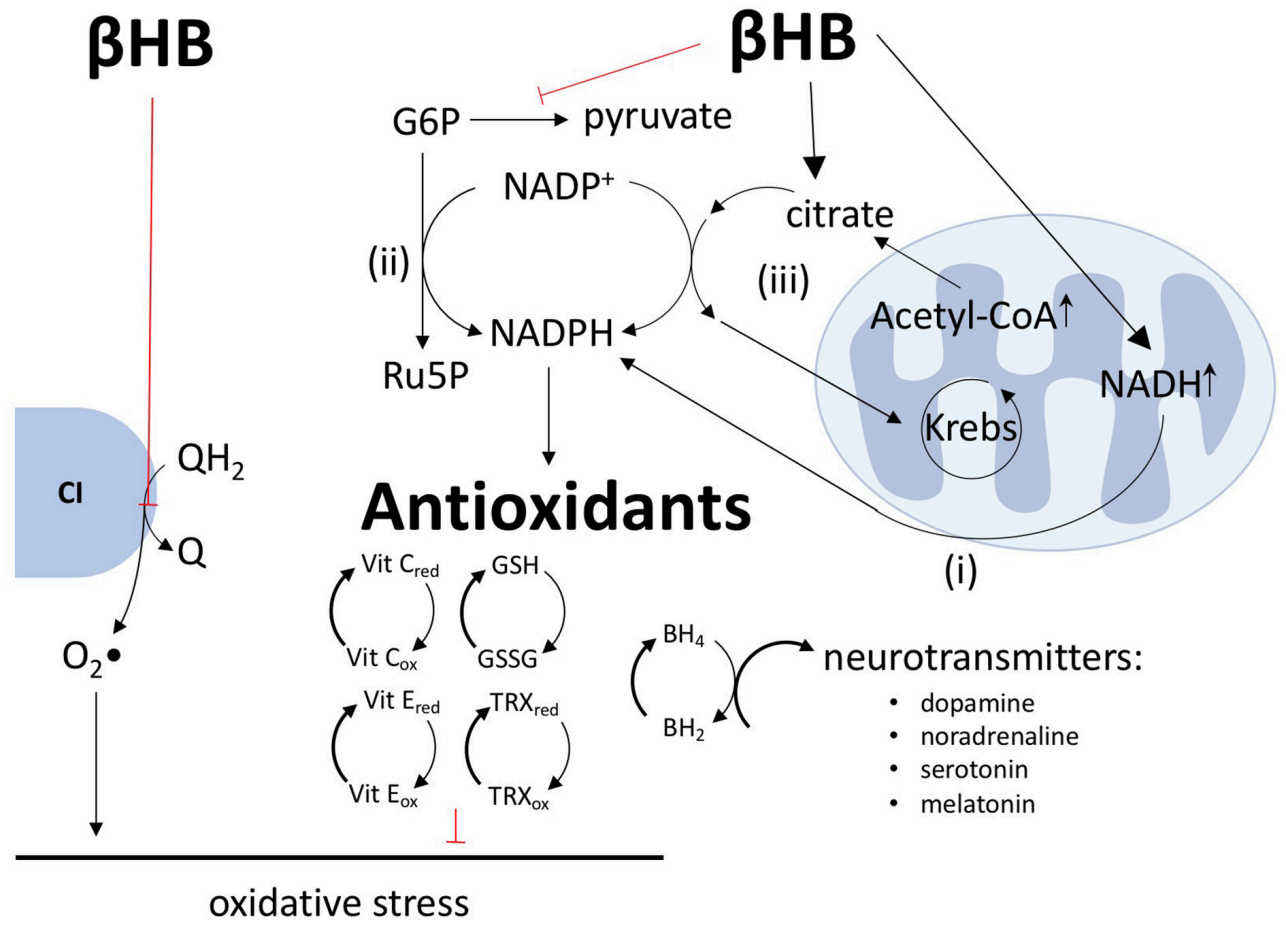
βHB decreases ROS production, increases antioxidant defenses, and increases neurotransmitter synthesis. βHB oxidizes the Q/QH_2_ couple to decrease the transfer of electrons from QH_2_ to oxygen by reverse electron transport at complex I (CI) and, thus, decrease the production of superoxide (O_2_•) radicals. βHB generates NADPH by (i) increasing the transfer of hydride ions from NADH to NADP^+^, (ii) inhibiting glycolysis, thus increasing pentose phosphate pathway flux, and (iii) increasing citrate-pyruvate and citrate-isocitrate cycle flux. NADPH, in turn, supports antioxidant defenses, including improving (reducing) the glutathione (GSH-GSSG), thioredoxin (TRX), and vitamins C and E reduced to oxidized ratios. NADPH reduces dihydrobiopterin (BH_2_) into tetrahydrobiopterin (BH_4_) and, thereby, increases the synthesis of the neurotransmitters dopamine, noradrenaline, serotonin, and melatonin.

By altering the ratios of redox couples within mitochondria and the cytoplasm, βHB metabolism may both diminish the production of reactive oxygen species (ROS) and bolster antioxidant defenses. Most mitochondrial ROS are generated via the process of reverse electron transport, in which electrons are passed from QH_2_ to oxygen at complex I to generate superoxide. Therefore, by oxidizing the Q/QH_2_ couple (8), βHB not only increases the production of ATP (see above), but also decreases the production of ROS (Figure 3).

In addition to increasing the Q/QH_2_ ratio and decreasing the NAD^+^/NADH ratio (8), βHB also decreases the NADP^+^/NADPH ratio through a variety of mechanisms. First, NADH and NADPH are tightly linked, especially in neurons, by the direct transfer of hydride ions from NADH to NADP^+^ via nicotinamide nucleotide transhydrogenase (12, 13). Second, by decreasing glycolytic flux, βHB forces glucose-6-phosphate down the pentose phosphate pathway, leading to the production of two equivalents of NADPH (8, 12–14). Third, by increasing ~15-fold the concentration of mitochondrial acetyl-CoA (8), βHB increases the concentration of mitochondrial citrate and the export of this citrate into the cytoplasm by the citrate-pyruvate and citrate-isocitrate cycles, each of which includes an NADP^+^ to NADPH reduction step catalyzed by malic enzyme and isocitrate dehydrogenase, respectively (12, 15). The products of these two carrier systems, oxaloacetate and α-ketoglutarate, are returned to the Krebs cycle to complete the circuit. Therefore, by increasing mitochondrial citrate concentrations, βHB forces the citrate-pyruvate and citrate-isocitrate wheels to spin faster to produce more NADPH (Figure 3). As all known intracellular antioxidants, either directly or indirectly, depend upon NADPH as the electron donor, βHB-mediated reduction of NADP^+^ into NADPH translates into an increase in the reduced levels of glutathione, thioredoxin, vitamins C and E, and other essential antioxidants (15).

By increasing NADPH levels, βHB may exert another beneficial effect in the context of PD: βHB may increase the synthesis of dopamine and other neurotransmitters. As the ultimate reducing agent, NADPH supports the reduction of dihydrobiopterin into tetrahydrobiopterin, a coenzyme critical in the synthesis of dopamine, noradrenalin, serotonin, and melatonin. Although the possible effect of βHB on these neurotransmitter systems is speculative and requires further investigation, it is worth mentioning if for no other reason than this mechanism by which βHB might increase neurotransmitter levels is distinct from the current standard of care for PD, levodopa therapy, which only provides the direct precursor to dopamine. Therefore, βHB may be able to increase the synthesis, not only of dopamine (3), but also of other neurotransmitters that are underproduced in PD and which contribute to the non-motor symptoms of PD not addressed by levodopa therapy (15).

βHB not only increases the Q/QH_2_ ratio to decrease the generation of ROS, and decreases the NADP^+^/NADPH ratio to increase antioxidant defenses and support neurotransmitter synthesis, but also has the unique ability to regulate all four of the “great” controlling nucleotide coenzyme couples: NAD^+^/NADH, NADP^+^/NADPH, acetyl-CoA/CoA, and ATP/ADP. These coenzymes diffuse throughout the cell, collectively regulating at least 1,500 known enzymatic reactions through their shared potential energies. Therefore, by regulating these four couples, βHB metabolism shapes the entire metabolome in ways that we are only beginning to appreciate and may affect not only energy crisis and oxidative stress, but also inflammation and apoptosis (15).

## G-Protein-Coupled Receptor (HCAR2) Activation

**Figure 4:**
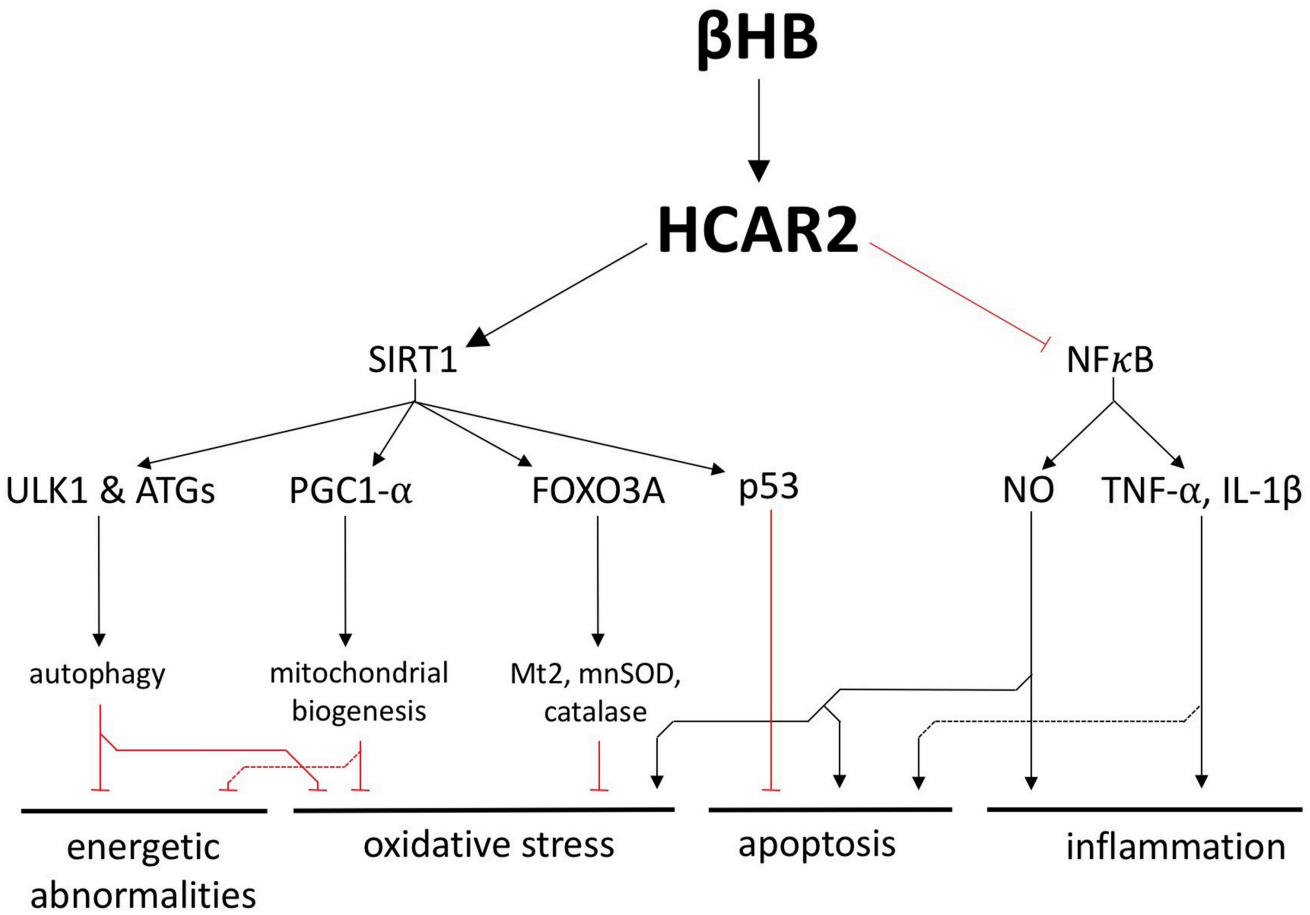
βHB exerts neuroprotective effects by activating hydroxycarboxylic acid receptor 2 (HCAR2). Activation of HCAR2 promotes the downstream activation of SIRT1 and inhibition of NFκB to protect against the fundamental pathologies of PD.

In addition to its role as a fuel substrate, βHB is a ligand for hydroxycarboxylic acid receptor 2 (HCAR2), a G-protein coupled receptor that is upregulated in the SN of PD patients (14, 16, 17). Although HCAR2 is upregulated in the PD brain, it is predicted to be underactive because PD patients also exhibit lower levels niacin, another HCAR2 ligand (17). Therefore, by substituting for niacin and activating upregulated HCAR2, βHB may be able to target a set of sensitized pathways in the PD brain, pathways that include the critical proteins SIRT1 and NFκB (Figure 4).

SIRT1 is a deacetylase whose activity is strongly associated with the generic health and longevity benefits of caloric restriction and that is underactive in the PD brain (18, 19). One way in which SIRT1 is thought to mediate its neurological benefits is by upregulating autophagy, a catabolic process active in all cells that facilities the degradation and recycling of damaged cellular components. SIRT1 activates a handful of autophagy proteins, including ULK1 and several ATGs, to increase the activity of this essential cellular recycling process and promote the clearance of defective and damaged mitochondria (20, 21). In a complementary fashion, SIRT1 activates the master regulator of mitochondrial biogenesis, PGC1-α, which is also underexpressed in, and has been strongly implicated in the pathogenesis of, PD (20, 22–24). Not only can SIRT1 increase autophagy and mitochondrial biogenesis, but it also induces FOXO3A-dependent expression of the antioxidant genes catalase, mnSOD, and Mt2 (20). Therefore, the stimulation of SIRT1 by βHB could protect cells against energy depletion and oxidative stress by improving the health of the mitochondrial pool and by bolstering antioxidant defenses. In addition, rodent studies suggest that a ketogenic diet induces SIRT1-dependent deacetylation of the transcription factor p53 and, thereby, increases the expression of anti-apoptotic proteins, decreases the expression of pro-apoptotic proteins, and ultimately protects neurons from apoptosis (25) (Figure 4).

By binding to HCAR2 on macrophages and microglia in the brain, βHB also inhibits NFκB-mediated neuroinflammation, a critical pathological feature in PD (26–28) (Figure 4). NFκB is a potent proinflammatory transcription factor that is elevated in the PD brain (29), has been proposed as a therapeutic target for PD (30), and induces the expression of TNF-α, IL-1β, and inducible nitric oxide synthase (iNOS) (31–33). In addition to stimulating an innate immune response in the brain, the cytokines TNF-α and IL-1β, which have likewise been proposed as targets for potential PD treatments (34), can also directly promote apoptosis by binding to death receptors on neurons (35, 36). The NO generated by iNOS, which is also pathologically important in PD (37), is likewise a proinflammatory molecule that exerts other pathological influences. NO can block glutamate reuptake, leading to excitotoxicity and apoptosis (32). NO can also provoke oxidative stress by post-translationally modifying proteins important in mitochondrial quality control and can induce nitroxidative stress by combining with superoxide to form peroxynitrite and other cytotoxic molecules (32).

## Histone Deacetylase Inhibition

It has been shown *in vitro* that histone deacetylase (HDAC) inhibition decreases α-synuclein toxicity and protects dopaminergic neurons from cell death (38). As a natural inhibitor of HDACs 1, 3, and 4 (16, 39), βHB may manage each of the pathologies underlying PD by regulating HDACs and altering gene expression.

**Figure 5:**
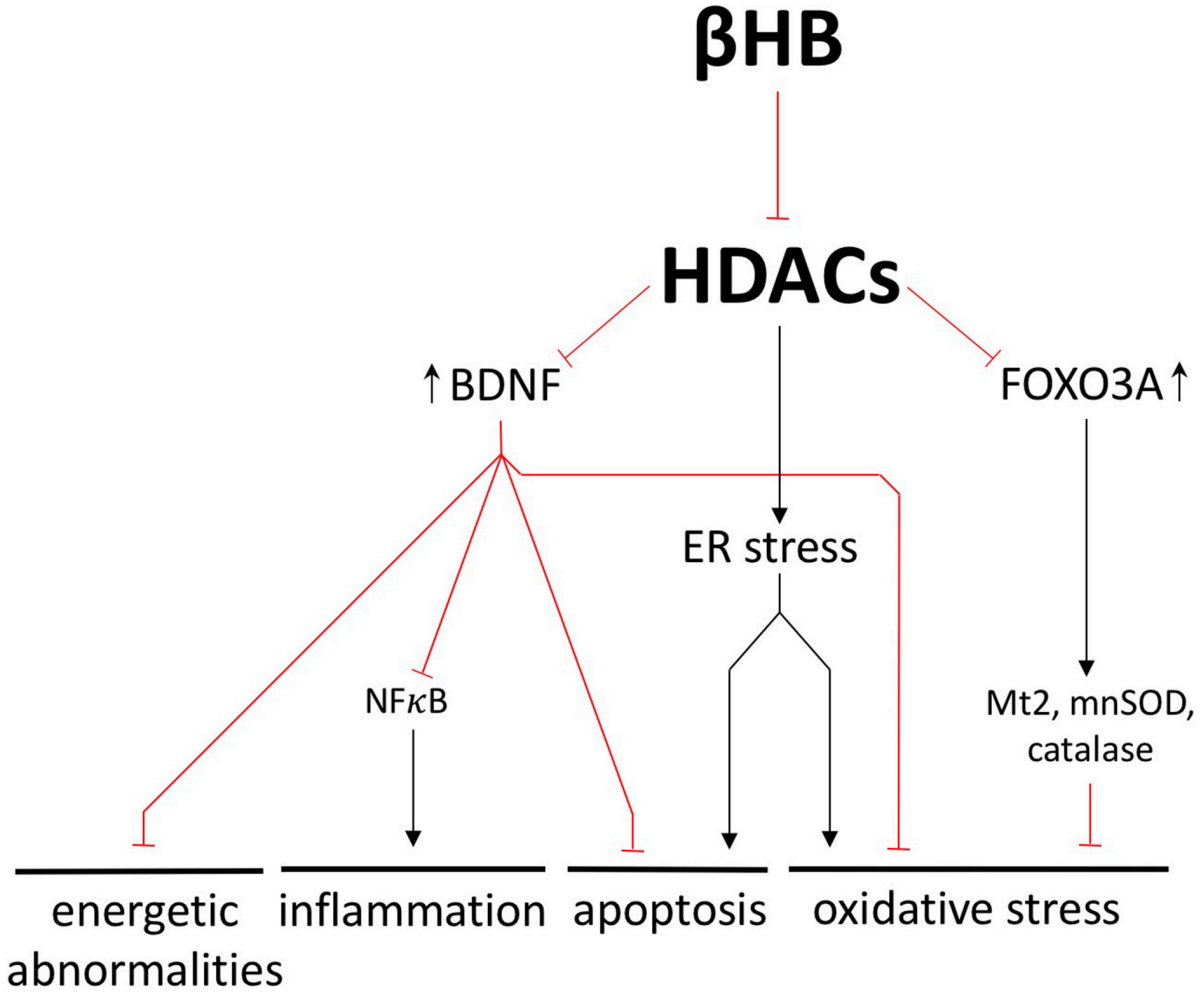
βHB exerts neuroprotective effects by inhibiting histone deacetylases (HDACs). βHB-mediated HDAC inhibition increases BDNF and FOXO3A expression and prevents endoplasmic reticulum (ER) stress. In these ways, βHB protects neurons against energetic abnormalities, inflammation, apoptosis, and oxidative stress.

For example, βHB-mediated HDAC inhibition in mice increases the expression of brain-derived neurotrophic factor (BDNF) (40), a molecule renowned for its putative ability to stimulate adult neurogenesis. In patients with PD, BDNF expression in the SN is significantly decreased (41), and, in rodent and primate models of PD, BDNF has been shown to protect dopaminergic SN neurons from cell death (42, 43). In mouse primary cortical neurons, βHB-mediated HDAC inhibition increases BDNF expression, decreases the NAD^+^/NADH ratio, and increases ATP (40, 44). BDNF also prevents NFκB-mediated neuroinflammation and apoptosis in rodent models of central nervous system inflammatory diseases (45, 46). Finally, BDNF increases the activity of multiple antioxidant enzymes and decreases oxidative damage in the basal ganglia of PD rats (47, 48). Therefore, by increasing BDNF expression alone, βHB may protect against energy depletion, oxidative stress, inflammation, and apoptosis (Figure 5).

BDNF is one representative example of how βHB-mediated HDAC inhibition can exert a wide range of neuroprotective effects by altering transcription; another example being βHB-mediated upregulation of the antioxidant genes catalase, mnSOD, and Mt2 (16, 49) (Figure 5). In fact, the βHB target, HDAC4, is responsible for the characteristic pattern of gene expression that differentiates PD neurons from healthy neurons (39). Furthermore, HDAC4 inhibition rescues gene expression in dopaminergic neurons derived from patients with PD. This genetic rescue, in turn, decreases endoplasmic reticulum stress, which is a cellular response to protein aggregation marked by (i) oxidative stress, (ii) apoptosis (50, 51), and (iii) glucocerebrosidase degradation (a critical autophagy-related enzyme already decreased in the PD brain) (52, 53) (Figure 5).

## Concluding Remarks

**Figure 6:**
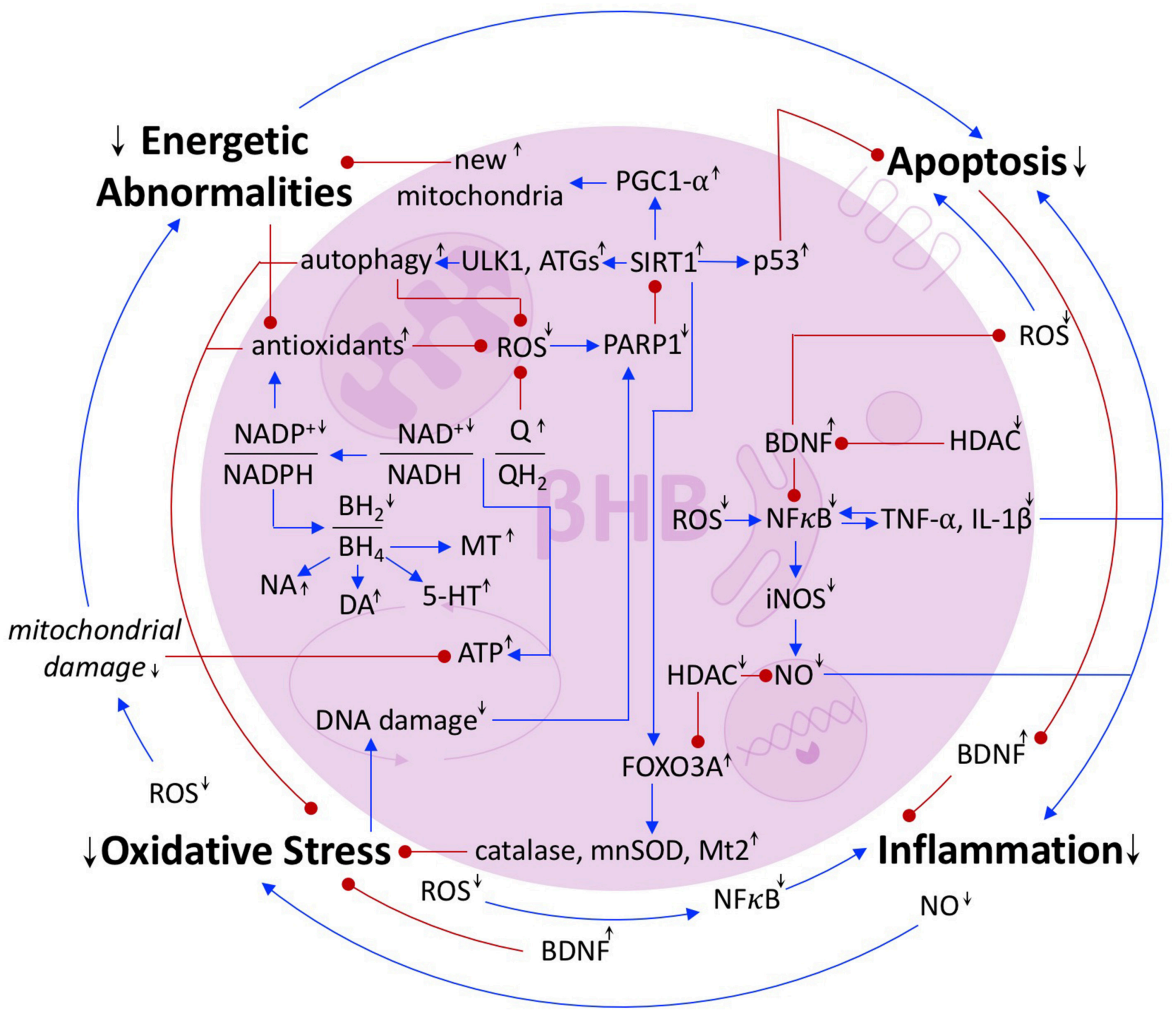
Summary of the interrelated pathologies of PD. Blue arrows represent activation or upregulation. Red dots represent inhibition or downregulation. Black up and down arrows represent the stimulatory or inhibitory effect of βHB on a given metabolite, ratio, protein, or process.

Segregating the pathologies underlying PD is, to some extent, an arbitrary exercise because they are deeply interconnected. Energetic abnormalities cause cells, which have been unable to maintain homeostasis, and thus may have compromised genomes, to undergo apoptosis (54). Apoptosis can deplete the extracellular pool of neuroprotective BDNF, thereby increasing inflammation (45, 46). The NO produced as part of the inflammatory response can induce oxidative stress (32), which can cause mitochondrial damage and increase the energy crisis (Figure 6, outermost loop).

Of course, PD pathology is not a simple loop, but a complex network. Oxidative stress induces DNA damage, exciting the activity of the DNA repair protein, PARP1, which catabolizes the SIRT1 cofactor, NAD^+^ (25), and thus precipitates further oxidative stress, energy crisis, and apoptosis. ROS also directly induces apoptosis and NFκB-mediated inflammation, the latter of which accentuates oxidative stress, causes apoptosis, and, because NFκB is induced by the cytokines that it induces, more inflammation (31) (Figure 6).

The pathologies that underlay PD are synergistic. This interconnectedness undermines any intervention aimed at a single point within the network. Perhaps that is why no true preventative treatments exist and why the current standard of care remains the largely palliative option of dopamine replacement therapy. However, there is a flipside to this complexity, for any molecule that can target many points within the network, as βHB can, may capitalize on the feedback loops, such that the multiple mechanisms by which it operates add up to more than the sum of their parts. In this review, we attempted to integrate the still largely independent bodies of literature on the biochemical effects of βHB and on that pathological mechanisms of PD in order to present a model for how βHB could be used to improve the multiple cellular pathologies of PD. This model, in combination with preliminary data that show the ketogenic diet may be ameliorative in PD (55), suggests that exogenous βHB, now available as an FDA-approved sports drink, may have therapeutic potential for the prevention and/or treatment of PD. Future studies, including those currently being conducted, will reveal the authenticity of this potential.

## Author Contributions

All authors listed have made a substantial, direct and intellectual contribution to the work, and approved it for publication.

### Conflict of Interest Statement

Intellectual property covering uses of dietary ketone and ketone ester supplementation is owned by BTG Ltd., the University of Oxford, the National Institute of Health and TΔS Ltd. Should royalties ever accrue from these patents, KC, as inventor, will receive a share of the royalties under the terms prescribed by the University of Oxford. KC is a director of TΔS Ltd., a company spun out of the University of Oxford to develop and commercialize products based on the science of ketone bodies in human nutrition. The remaining authors declare that the research was conducted in the absence of any commercial or financial relationships that could be construed as a potential conflict of interest.

## Abbreviations

ATGs: autophagy-related proteins
ATP: adenosine triphosphate
BDNF: brain-derived neurotrophic factor
BH_2_: dihydrobiopterin
BH_4_: tetrahydrobiopterin
β-HB: D-β-hydroxybutyrate
ER: endoplasmic reticulum
FOXO3A: forkhead box O3A
G6P: glucose-6-phosphate
HCAR2: hydroxycarboxylic acid receptor 2
HDAC: histone deacetylase
IL-1β: interleukin-1β
iNOS: inducible nitric oxide synthase
mnSOD: manganese superoxide dismutase
MPTP, 1-methyl-4-phenyl-1, 2, 3: 6-tetrahydropyridine
Mt2: metallothionein 2
NAD: nicotinamide adenine dinucleotide
NADP, nicotinamide adenine dinucleotide phosphate; NFκB: nuclear factor κ-light-chain-enhancer of activated B cells
NO: nitric oxide
O_2_•: superoxide
PARP1: poly ADP-ribose polymerase 1
PGC1-α: peroxisome proliferator-activated receptor gamma coactivator 1-α
PD: Parkinson's disease
^31^P-MRS: ^31^phosphorus-magnetic resonance spectroscopy
p53: tumor suppressor p53
Q: coenzyme Q
ROS: reactive oxygen species
Ru5P: ribulose-5-phosphate
SIRT1: silent mating type information regulation 2 homolog 1
SN: substantia nigra
TNF-α: tumor necrosis factor-α
TRX: thioredoxin
ULK1: unc-51 like autophagy activating kinase 1.
